# Neoadjuvant intra-arterial chemotherapy using an original four-lumen double-balloon catheter for locally advanced uterine cervical cancer

**DOI:** 10.18632/oncotarget.26518

**Published:** 2018-12-28

**Authors:** Tomohito Tanaka, Yoshito Terai, Satoe Fujiwara, Yoshimichi Tanaka, Hiroshi Sasaki, Satoshi Tsunetoh, Kazuhiro Yamamoto, Takashi Yamada, Masahide Ohmichi

**Affiliations:** ^1^ Department of Obstetrics and Gynecology, Osaka Medical College, Takatsuki, Japan; ^2^ Department of Radiology, Osaka Medical College, Takatsuki, Japan; ^3^ Department of Pathology, Osaka Medical College, Takatsuki, Japan

**Keywords:** uterine cervical cancer, locally advanced uterine cervical cancer, neoadjuvant chemotherapy, radical hysterectomy

## Abstract

**OBJECTIVE:**

We report a balloon-occluded arterial infusion therapy with an original four-lumen double-balloon catheter (4L-DB) which allows for the efficient injection of an anticancer agent at a high concentration to the target spot for patients with locally advanced uterine cervical cancer.

**METHODS:**

One hundred and forty-three patients with locally advanced cervical cancer treated with neoadjuvant intra-arterial chemotherapy (NAIAC) or a primary radical hysterectomy (PRH) were retrospectively assessed. The patients in the NAIAC group received irinotecan 70 mg/m2 intravenously on day 1 and 8 and cisplatin 70 mg/m2 intra-arterially using the 4L-DB on day 2 of a 21-day course, and two courses were performed in principle. The radical hysterectomy was performed within 6 weeks after NAIAC.

**RESULTS:**

Ninety-four patients were treated with NAIAC, and 49 patients undertook a PRH. The response rate of NAIAC on MRI was 92.6%. Fourteen patients (14.6%) had no evidence of cancer cells on pathologic diagnoses. The NAIAC group had a longer disease-free survival than the PRH group (p=0.02); however, the overall survival was not significantly different. The relative risk (RR) for recurrence was higher in patients with lymph node metastasis (RR, 4.31; 95% CI, 2.23-8.43) and lower in those who underwent NAIAC (RR, 0.30; 95% CI, 0.14-0.68).

**CONCLUSION:**

Our results with NAIAC using the 4L-DB catheter in locally advanced cervical cancer indicates beneficial effects on primary lesions and improves disease-free survival.

## INTRODUCTION

**Figure 1:**
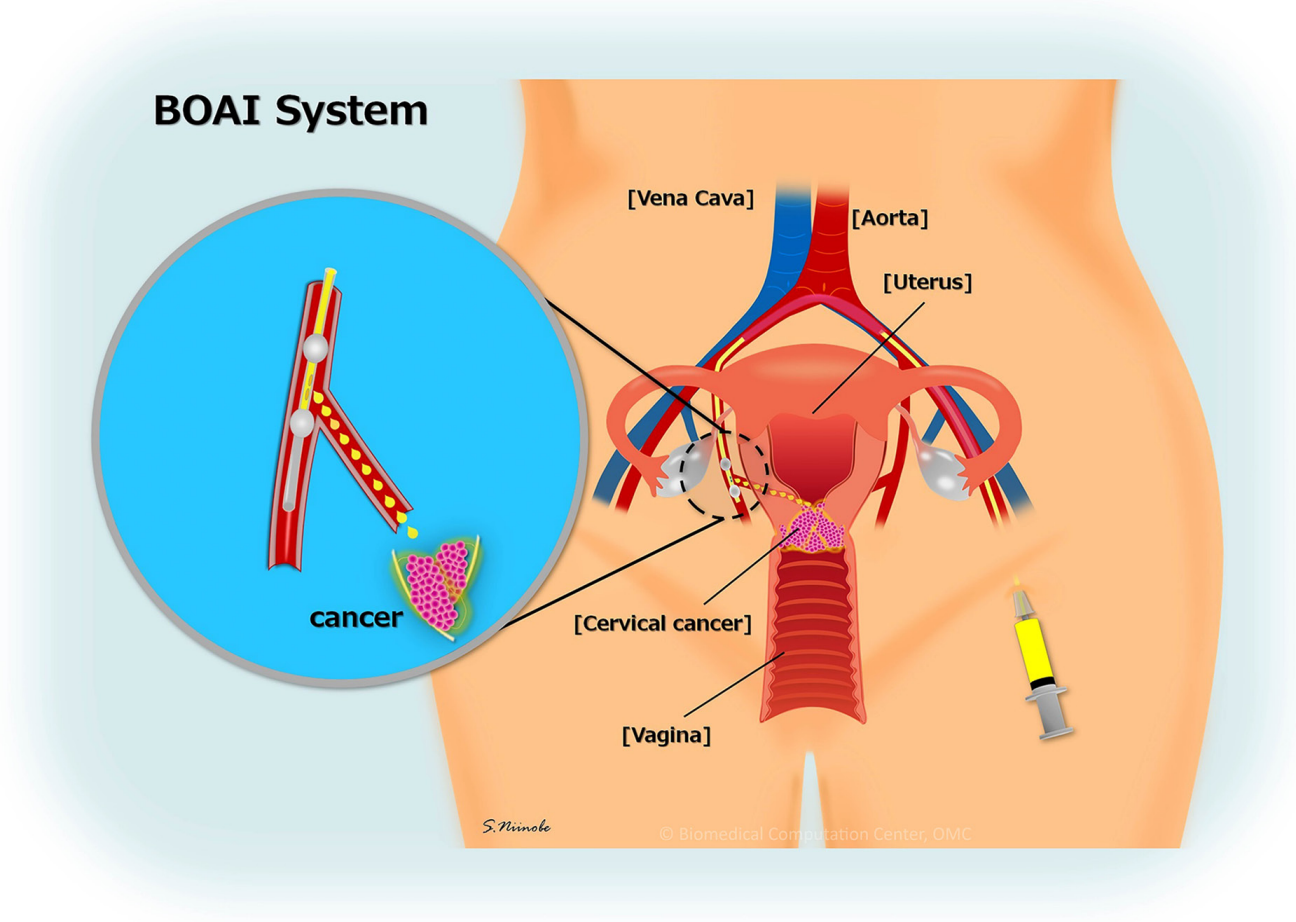
Balloon-occluded arterial infusion therapy (BOAI) Original four-lumen double-balloon (4L-DB) catheter has a double balloon on the end. A slit between the double balloon allows for the infusion of the anticancer drug locally. The catheter is inserted from the femoral artery. Through the opposite internal iliac artery, the end is placed on the superior gluteal artery. The anticancer drug injected through the slit remains between the double balloon and is delivered to the uterine artery or feeding artery selectively.

We previously reported a neoadjuvant intra-arterial chemotherapy (NAIAC) using an original four-lumen double-balloon (4L-DB) catheter (Figure 1) [1]. This catheter can expand the balloon in the internal iliac artery on the central and peripheral side of the uterine artery and selectively inject the anticancer agent into the uterine artery; the anticancer drug will not flow into other arteries, such as the inferior vesical artery, middle rectal artery, inferior gluteal artery or obturator artery. Therefore, a high concentration of the anticancer drug can be directly delivered to tumors through the uterine artery. The 4L-DB has also been used for bladder cancer [2–8], as well as cervical cancer [1]. Women with locally advanced cervical cancer (stage IB2, IIA2 and IIB) have a higher rate of recurrence and poor survival than those with early-stage disease (stage IA, IB1 and IIA1) [9]. In accord with the National Comprehensive Cancer Network's (NCCN) clinical guidelines, surgery or concurrent chemoradiotherapy (CCRT) is recommended in patients with stage IB1 and, in IIA1 cases, neoadjuvant chemotherapy (NAC) is not recommended [10] because a meta-analysis showed no advantage for NAC [11]. For patients with stage IB2, IIA2 and IIB disease, CCRT is also recommended, especially in patients with lymph node metastasis [10]. However, a NAC followed by radical hysterectomy is often commented on because several studies, including meta-analysis, have shown that NAC may improve the patient's prognosis [12, 13]. However, the appropriate treatment of stage IB2, IIA2 and IIB remains uncertain.

Adenocarcinoma currently comprises 10-20% of all cervical carcinomas in developed countries, compared to 5-10% three decades ago [14], and has a worse prognosis than squamous cell carcinoma [15, 16]. Several studies have shown that a radical hysterectomy has a better prognosis than radiotherapy in cervical adenocarcinoma [9, 17]. However, it may be difficult to perform a radical hysterectomy because cervical adenocarcinomas tend to form bulky tumors in some cases. NAC followed by a radical hysterectomy may improve the prognosis for bulky cervical adenocarcinoma. Cisplatin has been a key drug for cervical cancer because it has a 20% to 30% response rate as a single agent [18]. Irinotecan also has anticancer activity on cervical adenocarcinomas [19]. The response rate of intravenous neoadjuvant chemotherapy with cisplatin and irinotecan or nedaplatin and irinotecan for cervical cancer is 78.0-89.5% [20–23].

The current study demonstrates the efficacy and tolerance of NAIAC with cisplatin and irinotecan using a 4L-DB in patients with locally advanced cervical cancer, including adenocarcinoma.

## RESULTS

**Figure 2:**
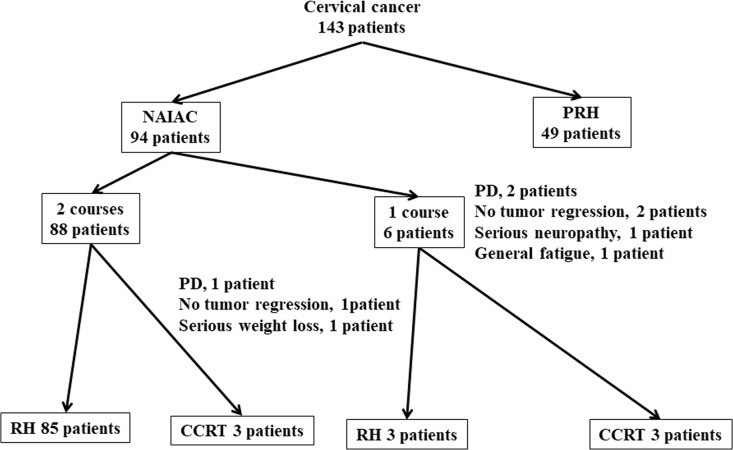
Among the 143 patients with locally advanced cervical cancer, 94 patients received neoadjuvant intraarterial chemotherapy (NAIAC), and 49 patients underwent primary radical hysterectomy (PRH) Among the 94 patients who receive NAIAC, 88 patients receive two courses of NAIAC. The other 6 patients receive one course of NAIAC because two patients had progressive disease (PD), one patient had no tumor regression, one patient had serious neuropathy, and one patient had general fatigue. Among the 88 patients who received two courses of NAIAC, 85 patients underwent radical hysterectomy (RH) after NAIAC. The other three patients receive CCRT after NAIAC because one patient had progression of the disease, one patient had no tumor regression, and one patient experienced serious weight loss. Among the six patients who received one course of NAIAC, three underwent RH, and three receive CCRT after NAIAC.

**Figure 3:**
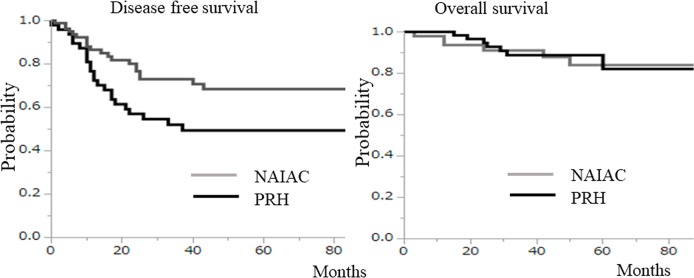
Those patients in the neoadjuvant intraarterial chemotherapy (NAIAC) group had a longer disease free survival rate than those in the primary radical hysterectomy (PRH) group (p=0. 02) However, overall survival was not signigicantly different between the two groups.

**Figure 4:**
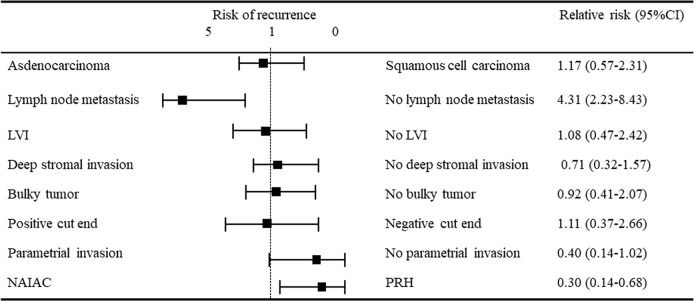
The results of multivariant analysis for risk of recurrence Lymph node metastasis and NAIAC were independent factors for recurrence. Other factors including histological type, lymphovascular involvement, deep stromal invasion (more than half myometrial invasion), bulky tumor (more than 4 cm), positive cut end, and parametrial invasion were not independent risk factors on multi variant analysis.

Among the 143 patients in the study with locally advanced cervical cancer, 94 patients received NAIAC, and 49 patients underwent primary radical hysterectomy (PRH) (Figure 2). Among the 94 patients who received NAIAC, 88 patients received two courses of NAIAC. The other 6 patients received one course of NAIAC because two patients had progression of the disease, one patient had no tumor regression, one patient had serious neuropathy, and one patient had general fatigue. Among the 88 patients who received two courses of NAIAC, 85 patients underwent radical hysterectomy (RH) after NAIAC. The other 3 patients received CCRT after NAIAC because one patient had progression of the disease, one patient had no tumor regression, and one patient experienced serious weight loss. Among the six patients who received one course of NAIAC, three underwent RH, and three received CCRT after NAIAC (Figure 1). Table 1 shows the characteristics, treatment and side effects of the 94 patients with locally advanced cervical cancer who underwent NAIAC. The mean (±standard deviation, SD) age of the patients was 49.4 ± 11.4 years. A total of 13 patients had the International Federation of Obstetricians and Gynecologists (FIGO) stage IB2 disease, 17 had stage IIA2 disease, and 64 had stage IIB disease. Histologically, 73 patients had squamous cell carcinoma, and 21 had adenocarcinoma. Eighty-eight patients underwent two courses of NAIAC, and the other six patients had only one course of NAIAC. Eighty-eight patients underwent RH after NAIAC and the other six patients received CCRT after NAIAC. The mean (± SD) tumor size was 46 ± 13 mm before NAIAC, decreasing to 14 ± 16 mm after NAIAC. The most frequent side effect was blood toxicity. Forty-six patients (48.9%) had grade 3 or grade 4 leukopenia. One patient (1.1%) had grade 3 thrombocytopenia. One (1.1%) had grade 3 anemia. There were no other grade 3 or more side effects. Table 2 shows the response to NAIAC for magnetic resonance imaging (MRI) and pathology. Among the 94 patients with locally advanced cervical cancer who underwent NAIAC, 37 patients had complete response (CR), 50 patients had partial response (PR), four patients had stable disease (SD), and three patients had progressive disease (PD) for MRI, resulting in a response rate of 92.6%. Among the 73 patients with squamous cell carcinoma who underwent NAIAC, 29 patients had CR, 39 patients had PR, three patients had SD, and two patients had PD, thus yielding a response rate of 93.2%. Among the 21 patients with adenocarcinoma who underwent NAIAC, eight patients had CR, 11 patients had PR, one patient had SD, and one patient had PD, resulting in a response rate of 90.5%. Pathologically, among the 88 patients with locally advanced cervical cancer who underwent NAIAC followed by RH, 14 patients (15.9%) had grade 3 response, 41 patients had grade 2 response, 23 had grade 1b response, 9 had grade 1a response, and one patient had grade 0 response. Among the 68 patients with squamous cell carcinoma, 13 patients (19.1%) had grade 3 response, 31 patients had grade 2 response, 18 had grade 1b response, five had grade 1a response and one patient had grade 0 response. Among the 20 patients with adenocarcinoma, one patient (5.0%) had grade 3 response, 10 patients had grade 2 response, five had grade 1b response, and four had grade 1a response. Table 3 shows the characteristics of patients who underwent NAIAC followed by RH and primary RH. Eighty-eight patients underwent NAIAC followed by RH. In contrast, 49 patients underwent primary RH. The mean (±standard deviation, SD) age was not significantly different between the groups (49.0 ± 11.4 vs. 50.5 ± 11.5 years, p=0.9). In the NAIAC group, 12 patients had FIGO stage IB2 disease, 17 had stage IIA2 disease, and 59 had stage IIB disease. In the PRH group, 15 patients had FIGO stage IB2 disease, 17 had stage IIA2 disease and 17 had stage IIB disease; the rate of IIB disease was significantly higher in the NAIAC group than that in the PRH group (67.0% vs. 34.7%, p<0.01). Histologically, 68 (77.3%) patients had squamous cell carcinoma, and 20 (22.7%) had adenocarcinoma in the NAIAC group. In contrast, 28 (57.1%) patients had squamous cell carcinoma, and 21 (42.9%) had adenocarcinoma in the PRH group; the rate of adenocarcinoma was significantly lower in the NAIAC group than that in the PRH group (22.7% vs. 42.9%, p=0.04). The mean pretreatment tumor size for MRI was not significantly different between the two groups (45 ± 12 vs. 44 ± 14 mm, p=0.6). In the NAIAC group, 27 patients underwent RT or CCRT, and 30 patients underwent chemotherapy after RH. The other 31 patients did not undergo adjuvant therapy. In the PRH group, 25 patients underwent RT or CCRT, and 19 patients underwent chemotherapy after RH. The other five patients did not undergo adjuvant therapy; the rate of no adjuvant therapy was lower in the NAIAC group than that in the PRH group (35.2% vs. 10.2%, p<0.01). One of 31 patients with no adjuvant therapy in the NAIAC group experienced recurrence on the vaginal stump. In contrast, two of five patients with no adjuvant therapy in the PR group had recurrence in the lung and pelvic cavity. Median follow up was shorter in the NAIAC group than that in the PRH group (30 vs. 48 months, p<0.01). The three-year disease free survival rate was significantly higher in the NAIAC group than that in the PRH group (64% vs. 52%, p=0.02). Figure 3 shows the disease free survival rate and overall survival rate for both groups. Those patients in the NAIAC group had a longer disease free survival than those in the PRH group (p=0.02). However, overall survival was not significantly different between the two groups. Figure 4 shows the results of multivariant analysis for the risk of recurrence. Lymph node metastasis (RR, 4.31; 95%CI, 2.23-8.43) and NAIAC (RR, 0.30; 95%CI 0.14-0.68) were independent factors for recurrence. Other factors, including histological type, lymphovascular involvement, deep stromal invasion (more than half myometrial invasion), bulky tumor (more than 4 cm), positive cut end and parametrial invasion, were not independent risk factors on multivariant analysis. Statistically, parametrial invasion was not an independent factor for recurrence (RR, 0.40; 95% CI, 0.14-1.02); however, this result suggested that those patients with parametrial invasion were less likely to experience recurrence. Most patients with risk factors including lymph node metastasis, LVI, deep stromal invasion, bulky tumor, positive cut end, and parametrial invasion received adjuvant chemotherapy or radiotherapy. Most patients with parametrial invasion received adjuvant radiotherapy, thus adjuvant radiotherapy may bring better DFS.

**Table 1:** Patients with locally advanced cervical cancer who received NAIAC

Total number of patients	94
Age (years old)	49.4 ± 11.4
FIGO stage	
IB2	13
IIA2	17
IIB	64
Histology	
Squamous cell carcinoma	73
Adenocarcinoma	21
NAIAC	
Two courses	88
One course	6
Treatment after chemotherapy	
Radical hysterectomy	88
CCRT	6
Tumor size	
Before NAIAC (mm)	46 ± 13
After NAIAC (mm)	14 ± 16
Side effect (grade3 or more)	
Leukopenia	46
Thrombocytopenia	1
Anemia	7

NAIAC, neoadjuvant intra-arterial chemotherapy; FIGO, The International Federation of Obstetricians and Gynecologists; CCRT, concurrent chemoradiotherapy.

**Table 2:** Response to NAIAC

Response to NAIAC							Response to NAIAC followed by RH					
	Number of patients	Response for MRI				RR	Number of patients	Pathological response				
		CR	PR	SD	PD			0	1a	1b	2	3
Total	94	37	50	4	3	92.6	88	1	9	23	41	14
Histological type												
SCC	73	29	39	3	2	93.2	68	1	5	18	31	13
AD	21	8	11	1	1	90.5	20	0	4	5	10	1

NAIAC, neoadjuvant intra-arterial chemotherapy; RH, radical hysterectomy; MRI, magnetic resonance imaging; SCC, squamous cell carcinoma; AD, adenocarcinoma; CR, complete response; PR, partial response; SD, stable disease; PD, progressive disease.

**Table 3:** Characteristics of patients with locally invasive cervical cancer who underwent a radical hysterectomy

	NAIAC (n=88)	Primary RH (n=49)	p value
Age (years old)	49.0 ± 11.4	50.5 ± 11.5	
FIGO stage			
IB2	12	15	0.04
IIA2	17	17	0.1
IIB	59	17	<0.01
Histology			
Squamous cell carcinoma	68	28	
Adenocarcinoma	20	21	0.04
Tumor size (mm)	45 ± 12	44 ± 14	0.6
Adjuvant therapy			
RT or CCRT	27	25	0.07
Chemotherapy	30	19	0.9
None	31	5	<0.01
Median follow up (months)	30	48	<0.01
3.y. disease free survival	64%	52%	0.02

NAIAC, neoadjuvant intra-arterial chemotherapy; RH, radical hysterectomy; FIGO, The International Federation of Obstetricians and Gynecologists, RT, radiotherapy; CCRT, concurrent chemoradiotherapy.

## DISCUSSION

NAIAC using a 4L-DB with intravenous irinotecan and intra-arterial cisplatin was feasible and effective on locally advanced cervical adenocarcinoma as well as squamous cell carcinoma. The NAIAC group had excellent DFS, compared with the PRH group. However, overall survival was not significantly different between the two groups. Moreover, the NAIAC group required less frequent adjuvant therapy than the PRH group.

The 4L-DB is an original catheter for intra-arterial chemotherapy which allows for a high concentration anticancer drug to be directly delivered to tumors through the uterine artery [1]. We previously reported NAIAC using a 4L-DB with platinum, mitomycin, and pirarubicin for cervical squamous cell carcinomas. The results revealed a 96.7% response rate with a 20% pathological CR [1]. The current study revealed a 93.2% RR with a 19.1% pathological CR for squamous cell carcinomas and a 90.5% PR with a 5% pathological CR for adenocarcinomas. The 4L-DB has been used not only for uterine cervical cancer but also for bladder cancer [2–8]. The Bladder preservation therapy combining intra-arterial chemotherapy using a 4L-DB and radiotherapy has already been performed.

There have been several literatures about NAC with platinum and CPT-11 for treatment of cervical cancer. Sugiyama et al. reported about NAC followed by a radical hysterectomy for stage of IB2 to IIIB cervical cancer. In this report, 23 patients were treated with intravenous CPT-11 (60 mg/m2, day1, 8 and 15) and cisplatin (60 mg/m2, day1) for 2 to 3 courses followed by a radical hysterectomy. The RR was 78% [20]. Syoji et al. also reported about NAC followed by a radical hysterectomy for stage of IB2 to IIIB cervical cancer. In this report, 42 patients were treated with intravenous CPT-11 (70 mg/m2, day1 and 8) and cisplatin (70 mg/m2, day1) for two courses followed by a radical hysterectomy. The RR in this report was 83.3% [24]. Moreover, Syoji et al. also compared neoadjuvant chemotherapy plus radical hysterectomy with radical hysterectomy alone in patients with stage II cervical squamous cell carcinoma presenting as a bulky mass. In this setting, there were no statistically significant differences between the two groups in operative time and the volume of intraoperative blood loss, and the patients in the NAC group were discharge earlier. The hazard ratio for disease-free survival (DFS) in the NAC group, as compared with that in the surgery alone group, was 0.36 (95% CI 0.08-0.91) [25]. Matsumura et al. reported about NAC follow by a radical hysterectomy for stage of IB2 to IIB cervical cancer. In this report, 48 patients were treated with intravenous CPT-11 (60 mg/m2, day 1 and 8) and nedaplatin (80 mg/m2, day 1) for 1 to 3 courses followed by a radical hysterectomy. The RR was 75.0% [23]. Yang et al. also reported about NAC follow by a radical hysterectomy for stage of IB2 to IIB cervical cancer. Among a total of 219 patients, 50 patients received intravenous irinotecan (60 mg/m2, day 1, 8 and 15) plus cisplatin (70 mg/m2, day 1) for two courses followed by a radical hysterectomy, 59 patients received intravenous paclitaxel (175 mg/m2, day1) plus cisplatin (70 mg/m2, day 1), and 110 patients received primary surgery. The RR in those patients who received irinotecan plus cisplatin was 67.3%, and survival analysis revealed no significant difference in disease-free-survival or OS between the NAC group and the primary surgery group [26]. In these literatures described above, anticancer agents were injected intravenously, and the RR was 67.3-83.3% [20, 23–26]. In our study, cisplatin was injected intra-arterialy using a 4L-DB, and the resulting RR was 92.6%, thus indicating that the RR in our study was higher than that in other previously published literatures. We believe that a high concentrate anticancer drug could be directly delivered to tumors through the uterine artery using a 4L-DB, thus yielding better results.

There have been several other literatures about intra-arterial chemotherapy. Gui et al. reported the comparison of intra-arterial and intravenous NAC in locally advanced cervical cancer. In the study, patients received three cycles of NAC every 3 weeks (cisplatin 70 mg/m2 on day 1, 5-fluouracil 1000 mg/m2 on day 1-4). Intra-arterial interventional chemotherapy was administered via right femoral artery catheterization. Each side of the uterine artery received half of the cisplatin dosage. The catheter was retained on the more severe side, maintaining a 24-hour infusion of 5-fluouracil for 4 consecutive days. The overall response rate was 84.9% vs. 88.2%, and the operability rate was 77.4% vs. 81.4% for intravenous vs. intra-arterial. There were no significant differences in toxicities, surgical duration, perioperative blood loss, and operative complications between the two groups. The intra-arterial group had a significantly lower parametrial infiltration for postoperative pathological examination. Moreover, the positive vaginal margin, lymph node metastasis and intravascular tumor embolism showed no significant differences. The recurrence, distant metastasis, and 5-year survival rates did not show any significant differences between the two groups [27]. Wen et al. reported a prospective randomized controlled study on multiple neoadjuvant treatment for patients with stage IB2 to IIA cervical cancer. One hundred and twenty-three patients were enrolled and randomly assigned to receive one of the following four treatments: radical surgery, brachytherapy with a total dose of 5Gy to point A followed by radical surgery, intravenous chemotherapy with cisplatin 50 mg/m2 plus 5-fluoroucil 750 mg/m2 at a 2-weeks interval for two courses followed by radical surgery, or intra-arterial chemotherapy with the same regimen as the intravenous chemotherapy followed by radical surgery. The clinical overall response rates were 61.3%, 42.9% and 79.3% in brachytherapy, intravenous chemotherapy and intra-arterial chemotherapy, respectively. The 3-year progression-free survival rates were 70.7%, 66.3%, 81.5% and 79.7% in surgery alone, brachytherapy, intravenous chemotherapy and intra-arterial chemotherapy, respectively. Three-year overall survival rates were 73.3%, 68.3%, 82.9% and 80.4% in the surgery alone, brachytherapy, intravenous chemotherapy and intra-arterial chemotherapy, respectively. Multivariate analysis also showed that only lymph node status correlated with progression-free survival [28]. Tsubamoto et al. reported on neoadjuvant transuterine arterial chemotherapy followed by radical hysterectomy in patients with locally advanced cervical cancer. Seventy-three patients received transuterine arterial chemotherapy combined with intravenous nedaplatin, irinotecan, paclitaxel, or etoposide administration. The radiological response rate was 96%. Multivariate analysis revealed that a tumor size of more than 60 mm and lymph node metastasis were negative prognostic factors for overall survival. Intra-arterial chemotherapy is still controversial and is not convenient compared to intravenous chemotherapy. However, several literatures have shown that intra-arterial chemotherapy had a higher response rate than the intravenous chemotherapy described above. Moreover, intra-arterial chemotherapy using a 4L-DB could deliver a high concentration of anticancer drug to the tumor - in theory. In our previous report about intra-arterial chemotherapy using a 4L-DB, the platinum concentration was significantly higher in the tumors with a CR to NAIAC than those with a PR and SD (p<0.0001, CR; 11.5 ± 0.8 μg/g, PR; 7.3 ± 0.5μg/g, SD; 4.5 ± 0.1 μg/g) [1]. Therefore, intra-arterial chemotherapy, especially via a 4L-DB, may be inconvenient. However, it may have advantages compared with intravenous chemotherapy.

Making comparison between NAC plus radical surgery and primary radiotherapy, a meta-analysis of randomized trials including 872 patients with locally advanced cervical cancer showed that NAC obtained a better DFS (HR = 0.68, 95% CI = 0.56-0.82) and OS (HR = 0.65, 95% CI = 0.53-0.80) [29] [29]. A recent study by Gupta et al. indicated that cisplatin-based CCRT resulted in superior DFS compared with NAC followed by radical surgery in locally advanced cervical cancer. OS was not significantly different between the two groups. In the subgroup analysis, DFS was significantly inferior in the NAC plus surgery group in stage IIB disease. In stage IB2 and II disease, DFS was not significantly different between the NAC and CCRT groups. However, a lot of patient in this study did not receive complete surgery. 21.5% of patients crossed over (presurgery crossover and intraoperative unresectable disease) to receive definitive CCRT [30]. In our study, only 6 patients (6.4%) receive presurgical CCRT. Furthermore, all patients who underwent radical surgery received complete surgery.

Meta-analysis showed that NAC followed by radical surgery had no prognostic advantage in patients with stage 1B1 to IIA cervical cancer, compared to primary radical surgery. NAC was related with a lower rate of tumor size and lymph node metastasis than primary radical surgery. Furthermore, NAC reduced the need for adjuvant radiotherapy and decreased the rate of distant metastasis [11]. The multicenter retrospective study showed that recurrence-free survival was significantly longer in stage IB2 to IIB patients who achieved an overall optimal response to NAC than in those who did not. The authors concluded that optimal responders after NAC followed by radical surgery did not need further treatment. Although this study compared responders with no responders who underwent NAC followed by radical surgery, the sensitivity for NAC may be an important prognostic factor [12]. Other meta-analysis also showed the sensitivity of NAC could be an important prognostic factor in patients with stage IB1 to IV cervical cancer [13]. JCOG0102, which is a prospective randomized control trial for NAC in stage IB1 to IIB cervical cancer in Japan, showed that NAC had no prognostic advantage; however, it did reduce the need for adjuvant radiotherapy [31]. In our study, DFS was significantly longer in those patients who received NAIAC than in those who underwent primary radical surgery. Furthermore, NAIAC was an independent prognostic factor in multivariant analysis, and 35% patients who received NAIAC did not need any adjuvant therapy. Although further examination is needed, we believe that our 4L-DB could provide excellent results.

This study is associated with several important limitations that may potentially decrease its value. First, the sample size was not large enough for complete analysis. Second, there was selection bias in choosing the primary treatment, as the surgeon tended to choose NAIAC when they felt that it would be difficult to perform a radical hysterectomy due to a large tumor size or parametrial invasion. Third, the results were conducted through those patients who underwent a radical hysterectomy; patients who underwent NAIAC followed by radiotherapy were not considered. Fourth, we did not compare NAIAC plus radical surgery to CCRT. From this point of view, the conclusion is not definitive.

In conclusion, NAIAC using a 4L-DB with intravenous irinotecan and intra-arterial cisplatin is feasible and effective on locally advanced cervical cancer. The NAIAC group had an excellent DFS, as opposed to that in the PRH group. However, overall survival was not significantly different between the groups. The NAIAC group, as well, needed less frequent adjuvant therapy than the PRH group. Therefore, NAIAC using a 4L-DB with intravenous irinotecan and intra-arterial cisplatin followed by radical hysterectomy might be a useful strategy for locally advanced cervical cancer.

## MATERIALS AND METHODS

### Participants

The present study included 143 Japanese patients with cervical cancer who were treated at Osaka Medical College between April 2006 and April 2017. Patients were eligible for inclusion in the study when they met the following criteria: (1) patients with an International Federation of Obstetricians and Gynecologists (FIGO) stage IB2, IIA2 or IIB cervical squamous cell carcinoma or adenocarcinoma who were treated by NAIAC with cisplatin and irinotecan using a 4L-DB or a radical hysterectomy with salpingo-oophorectomy as an initial treatment. The surgeon chose NAIAC when the patients had a seriously restricted uterus for cancer invasion. In contrast, PRH was performed when the patient had no seriously restricted uterus; (2) age less than 70 years; (3) a World Health Organization (WHO) performance status of 0 to 2; (4) fulfillment of pretreatment laboratory requirements, including a leukocyte count more than 3000/mm3, a platelet count more than 100 000/mm3, serum creatinine less than 1.5 mg/dl, serum bilirubin less than 1.5 mg/dl, and normal serum aspartate transaminase (AST) and serum alanine transaminase (ALT); (5) patients without any other major organ disease; and (6) the patient had sufficient clinical data regarding the oncologic outcome, including the date of recurrence. All patients were staged according to FIGO criteria, and the histological subtype was assigned according to the criteria of the WHO classification. The present study was approved by the institutional review board (IRB) of Osaka Medical College. Written informed consent was obtained from all patients for NAIAC and for the use of clinical records in the present study. Those patients who underwent a primary radical hysterectomy provided their written informed consent at the time of the primary surgery to use their clinical records for an IRB-approved study, and the IRB approved this consent procedure.

### Intra-arterial chemotherapy

For intra-arterial infusion therapy, we developed an original 4L-DB catheter (Clinical Supply Japan) for the simple and efficient injection of an anticancer agent at a high concentration to target spots in patients with advanced uterine cervical cancer [1]. Under local anesthesia, following Seldinger's technique, polyethylene catheters of 6-French diameter were inserted through both femoral arteries. Each catheter tip was placed in the internal iliac artery. While the guidewire was detained in the peripheral artery of the internal iliac artery, the catheter was passed through the junction of the uterine artery, which was the target vessel, just distal to the branching out of the superior gluteal artery. To confirm the correct position of the catheter and effective perfusion, pelvic arteriography was performed during catheterization procedures. Each time after the completion of treatment, the catheters were removed, and sandbags were used to apply firm pressure over each groin area for 6h. The regimen included the following: intravenous irinotecan (CPT-11) (70 mg/m2, day1 and 8) and intra-arterial cisplatin (75 mg/m2, day2) using the 4L-DB for two courses every 21 days. Cisplatin was administered intra-arterially within 30min in divided doses via the bilateral internal iliac arteries. Hydration with normal saline and 5% dextrose began 3h before chemotherapy, with careful monitoring of urine volume.

### Treatment response

Complete blood cell counts and renal and hepatic function tests were repeated before each course. Toxicity was graded according to the WHO criteria. The tumor was monitored with magnetic resonance imaging (MRI; SIGNA MR/i; GE, Slough, UK). Response was measured as the product of the two largest perpendicular diameters of the cervical mass lesion. Patients were evaluated for response with a physical examination and MRI after two courses of therapy. A CR was defined as the complete disappearance of all clinically detectable disease. A 50% or more decrease in tumor size constituted a partial PR. A SD was defined as no significant change, and a PD was defined as a more than 25% increase in tumor size or the appearance of new lesions or hydronephrosis.

In addition, histological changes were also evaluated in surgical specimens using the following criteria set by the Japan Society for Cancer Therapy: grade 0 was defined as the absence of degenerative or necrotic change after chemotherapy; grade 1a was defined as degeneration, necrosis, or cytolysis in less than one-third of the cancer cells; grade 1b was defined as degeneration, necrosis, or cytolysis in more than one-third but less than two-thirds of the cancer cells; grade 2 was defined as remarkable degeneration, necrosis, cytolysis, or the disappearance of cancer cells in more than two-thirds of the cancer cells; grade 3 was defined as all cancer cells becoming necrotic, the occurrence of cytolysis, or the presence of granulomatous tissue or fibrosis.

### Local therapy

Following NAIAC, a radical hysterectomy with pelvic lymphadenectomy was performed on patients responding to the NAIAC, if possible. After surgery, patients with any poor prognostic factors, including lymph node metastasis, parametrial infiltration, vaginal invasion, or ovarian metastasis received postoperative adjuvant radiotherapy (RT) [Linac, 40 to 50 Gy; and/or RALS (Remote After Loading System), 30 to 40 Gy] or chemotherapy. Patients who did not respond to NAIC received CCRT, which was external-beam radiation (Linac, 50–60 Gy) with Cisplatin 40 mg/m2/week and brachytherapy delivered by remote control after loading the system using 60CO (RALS, 40 to 50 Gy).

### Statistical analysis

All of the statistical analyses were performed using the JMP software package (version. 11.1.1). Continuous variables are expressed as the mean ± standard deviation. The Student T-test was used to compare continuous variables, and Pearson's Chi-square test was used to compare frequencies. Overall survival curves and DFS curves were plotted according to the Kaplan-Meier method and were analyzed by the log-rank test. Relative risk was calculated by Cox's proportional hazards model. P values of <0.05 were considered to indicate statistical significance.

## Abbreviations

4L-DB: four-lumen double-balloon catheter
NAIAC: neoadjuvant intra-arterial chemotherapy
PRH: primary radical hysterectomy
RH: radical hysterectomy
RR: relative risk
NCCN: National Comprehensive Cancer Network's
NAC: neoadjuvant chemotherapy
CCRT: concurrent chemoradiotherapy
RT: radiotherapy
SD: standard deviation
FIGO: International Federation of Obstetricians and Gynecologists
CR: complete response
PR: partial response
SD: stable disease
PD: progressive disease
DFS: disease-free survival
